# β-lactolin increases cerebral blood flow in dorsolateral prefrontal cortex in healthy adults: a randomized controlled trial

**DOI:** 10.18632/aging.103951

**Published:** 2020-09-29

**Authors:** Yasuhisa Ano, Keiko Kobayashi, Mamoru Hanyuda, Ryuta Kawashima

**Affiliations:** 1 Kirin Central Research Institute, Kirin Holdings Company, Ltd., Kanazawa-ku, Yokohama 236-0004, Japan; 2 Breast Health Clinic, Chuo-ku, Tokyo 103-0025, Japan; 3 Institute of Development, Aging and Cancer (IDAC), Tohoku University, Aoba-ku, Sendai 980-0872, Japan; 4 NeU Corporation, Chiyoda-ku, Tokyo 101-0048, Japan

**Keywords:** β-lactolin, β-lactopeptide, cerebral blood flow, dorsolateral prefrontal cortex, near-infrared spectroscopy (NIRS)

## Abstract

The number of elderly individuals with age-related cognitive decline or dementia is rapidly increasing. Dairy product consumption, including β-lactolin, is beneficial for their cognitive function. The underlying mechanism of β-lactolin’s effects on human brain activity is yet to be investigated. We examined the β-lactolin effects on human cerebral blood flow (CBF) using near-infrared spectroscopy (NIRS) in a placebo-controlled randomized double-blind study, which reported according to the CONSORT guidelines. Fifty healthy participants (aged 45–60 years) were randomly allocated into the β-lactolin or the placebo group (n = 25 each) and supplemented for 6 weeks. During the 6^th^ week, oxy-hemoglobin during the working memory tasks was measured using 34-channels (CHs) NIRS. The changes of oxy-hemoglobin, which represents the CBF, in CH 23 located at the left dorsolateral prefrontal cortex (DLPFC) during the spatial working memory task showed higher statistical significance (false discovery rate (*q*) = 0.045) in the β-lactolin than in the placebo group. The oxy-Hb changes in CH23 have a co-relationship with the working memory task reaction time. This clinical trial showed an increase in the CBF in the left DLPFC area during the 6-week β-lactolin supplementation. This study contributes to elucidating the underlying mechanisms of β-lactolin on cognitive performance.

## INTRODUCTION

The global number of people with age-related cognitive decline or dementia is rapidly increasing, due to the growing aging population. Previous epidemiological studies have shown that daily consumption of dairy products prevents age-related cognitive decline [1–4]. Some studies have demonstrated that dairy products prevent cognitive decline in rodents and humans [5, 6]. Indeed, camembert cheese, a dairy product fermented with fungi, prevents the development of Alzheimer’s disease in a transgenic mouse model [7]. Our group identified β-lactopeptide, tryptophan-tyrosine (WY)-related lactopeptides including WY and glycine-threonine-tryptophan-tyrosine (GTWY), which improve cognitive and mood function and prevent Alzheimer’s disease pathology [5, 8, 9]. Especially, β-lactolin of GTWY peptide derived from β-lactoglobulin is rich in fermented dairy products including camembert cheese, blue cheese, and whey enzymatic digestions [5, 10]. Orally administered β-lactolin is delivered to the brain and inhibits the activity of monoamine oxidase B (MAO-B) in mice, thus, increasing the dopamine levels in the cortex and the hippocampus [11, 12]. β-lactolin improves spatial working and episodic object recognition memory in pharmacologically induced amnesia in young and aged mice [5]. β-lactolin also improves memory impairment and suppresses Alzheimer’s pathologies including Aβ-deposition and inflammation in the brain in Alzheimer’s transgenic 5×FAD mice [13].

In addition to preclinical studies’ demonstrations, our previous clinical study showed that supplementation with β-lactolin for 6 weeks improves the score of the verbal fluency and Stroop tests, evaluating the executive function and the inhibition of attention in healthy middle-aged adults, respectively, compared with the placebo group in a randomized placebo-controlled trial [14]. We also demonstrated that the supplementation with β-lactolin improves selective and sustained attention (evaluated by a visual-cancellation test) and the working memory (evaluated by paired-associate learning tests in healthy older adults) compared with the placebo group in a randomized placebo-controlled trial [6]. These previous demonstrations showed that supplementing with β-lactolin improves attention, executive function, and memory retrieval especially associated with the function of the dorsolateral prefrontal cortex (DLPFC). The DLPFC is an area in the prefrontal cortex of the brain, which is essential for the executive function, such as working memory, inhibition, cognitive flexibility, planning, and abstract reasoning. Thus, the activation of DLPFC is associated with these cognitive functions [15–17]. The impairment of the DLPFC’s function is associated with cognitive decline in elderly patients and those with dementia; therefore, supplementation of β-lactolin might be expected to prevent cognitive decline and dementia. Conversely, the association of β-lactolin supplementation with DLPFC activation is yet to be evaluated.

Prefrontal cortex cerebral nerve activity is evaluated using magnetic resonance imaging (MRI), single-photon emission computed tomography (SPECT), and near-infrared spectroscopy (NIRS). NIRS is a non-invasive and easy-to-use evaluation; therefore, the number of clinical trials using NIRS to measure the DLPFC’s function has been increasing in healthy participants [18]. Compared with MRI and SPECT, NIRS measures the regional cerebral blood flow (rCBF) on the cortical surface. The region of the DLPFC, activated by β-lactolin supplementation, is the superficial cortex, of which the rCBF is detectable by the NIRS. Recent studies using NIRS have assessed cortical activation and functional connectivity during a visuospatial working memory task and auditory working memory, measuring the activity of the DLPFC during memory encoding and retrieval [19–21]. These demonstrations showed that the measurement using NIRS is available for the evaluation of the DLPFC activity.

In this study, we conducted a randomized placebo-controlled trial and measured the DLPFC’s activity during cognitive tasks to evaluate the effects of β-lactolin on the rCBF in the DLPFC. We measured the oxy-hemoglobin (oxy-Hb) levels during the working memory tasks by multi-channel (CH) 34CH NIRS at 0 and 6 weeks of the interventions to evaluate the DLPFC’s activation. To elucidate the underlying mechanism of β-lactolin on the previously demonstrated cognitive function, we measured the rCBF in healthy older adults, similar to the previously performed works. These demonstrations would contribute to elucidate the associations between the DLPFC’s activation and the rCBF with improved cognitive function by the supplementation of β-lactolin.

## RESULTS

### Baseline characteristics of the study group

**Figure 1:**
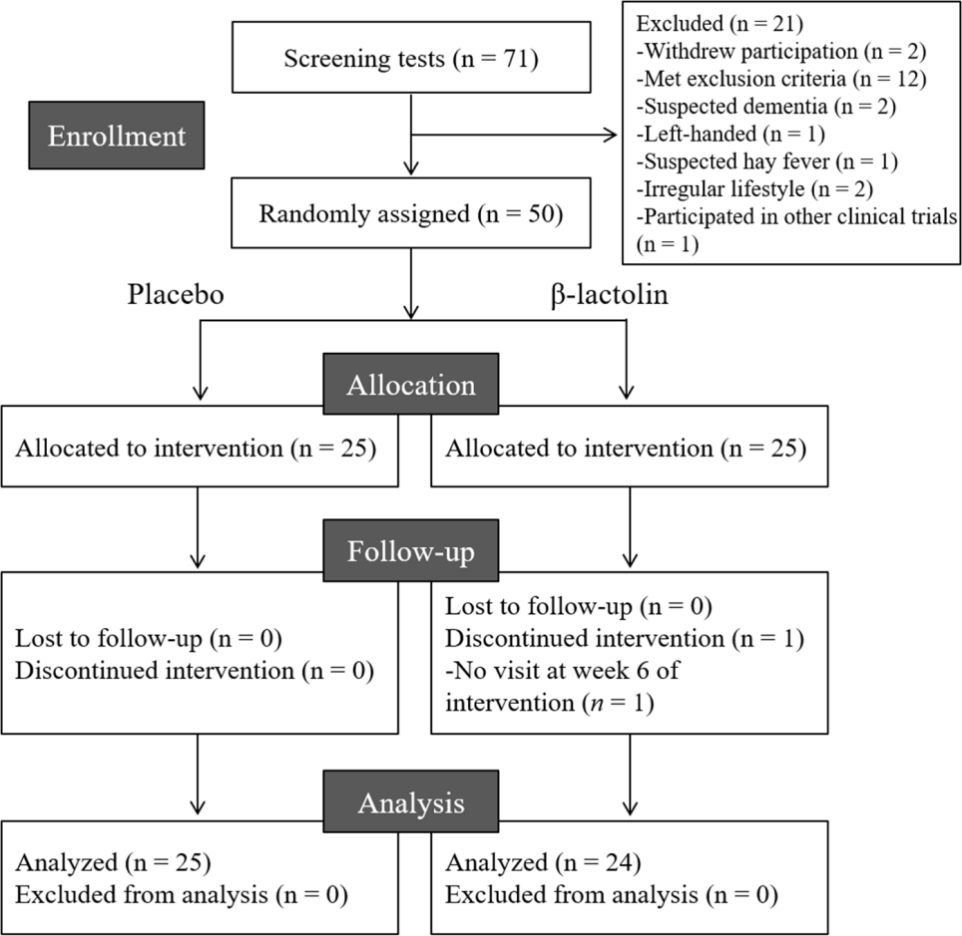
CONSORT diagram. We included 50 of the 71 screened participants in this study; they were randomly allocated to the placebo (n = 25) and β-lactolin (n = 25) groups. One participant dropped out and, therefore, the data of 25 and 24 participants in the placebo and β-lactolin groups were analyzed, respectively.

The flow chart of the participant selection process is presented in Figure 1. We recruited 71 Japanese-speaking healthy older adults aged between 45–60 years (male/female = 35/36). Following screening, 50 participants (male/female = 26/24) were included and 21 participants were excluded as they withdrew their participation (n = 2), met the exclusion criteria (n = 12), had suspended dementia (n = 2), were left-handed (n = 1), had suspected hay fever (n = 1) and irregular lifestyle (n = 2), and participated in other clinical trials (n = 1). The 50 participants were randomly allocated to the β-lactolin and placebo groups and were supplemented for 6 weeks. One participant in the β-lactolin group did not visit at week 6 of the intervention period and excluded. Finally, the data of 24 (male/female = 13/11) and 25 participants (male/female = 13/12) in the β-lactolin and the placebo group, respectively, were analyzed (Table 1).

**Table 1:** Participants’ characteristics at baseline.

**Characteristics**	**Placebo**	**β-lactolin**	***p***
Age	52.2 ± 4.3	52.2 ± 4.9	0.996
Male/Female	13 / 12	13 / 11	0.879
BMI	22.7 ± 4.1	22.9 ± 3.7	0.894
MMSE	29.3 ± 1.1	29.0 ± 1.1	0.457

Data are presented as means ± SD: the placebo (n = 25) and the β-lactolin (n = 24). *p-*values were calculated using unpaired t-tests, except for those for the male/female ratios, who were calculated using χ^2^ tests.

BMI, body mass index; MMSE, Mini Mental State Examination; SD, standard deviation.

### rCBF measured using a 34CH NIRS (Primary outcome)

**Figure 2:**
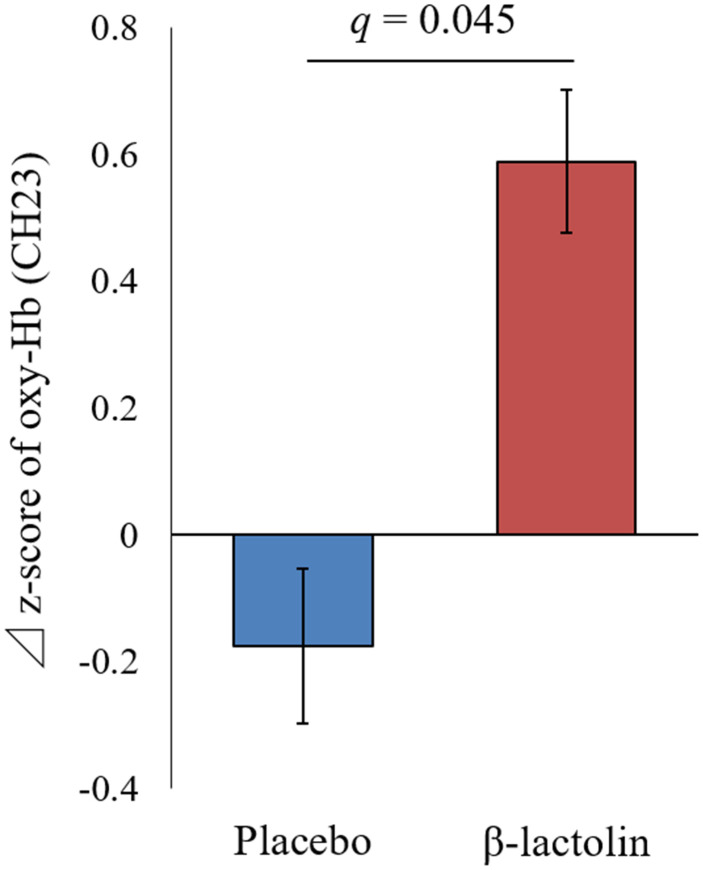
**Changes of the oxy-Hb levels during spatial working memory in the left DLPFC.** Oxy-Hb measurement at weeks 0 and 6 and changes between weeks 0 and 6 in CH23 during the spatial working memory task. Data are presented as means and ± standard errors for the placebo (n = 25) and the β-lactolin group (n = 24). Group differences were identified using ANOVA and FDR correlation. A value *q* < 0.05 considered statistically significant. ANOVA, analysis of variance; CH, channel; Hb, hemoglobin; FDR, false discovery rate; DLPFC, dorsolateral prefrontal cortex

**Figure 3:**
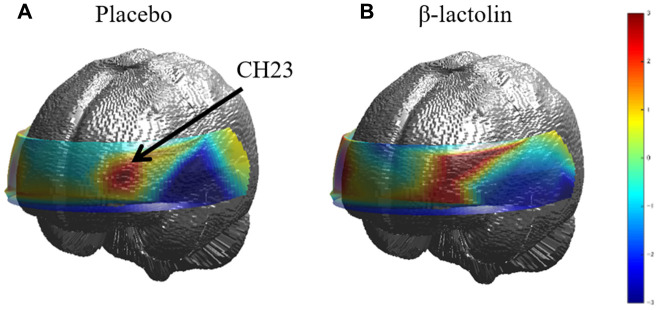
**Topographic images of oxy-Hb during spatial working memory.** (**A** and **B**) The topographic maps during the spatial working memory task for oxy-Hb reveal the distribution of rCBF at week 6 of the intervention for both groups (placebo and β-lactolin, respectively). Hb, hemoglobin; rCBF, regional cerebral blood flow.

At intervention weeks 0 and 6, the rCBF during working memory tasks was measured by the 34CH NIRS. The changes in the mean z-score of oxy-Hb for each task at the region of interest (ROI) are shown in Tables 2 and 3 and in Supplementary Tables 1 and 2. The baseline oxy-Hb levels in CH11, CH13, CH14, CH20, CH22, and CH23 at week 0 of the intervention did not differ between the groups (analysis of variance [ANOVA]; *q* = 0.456, 0.701, 0.456, 0.701, 0.456, and 0.456 [verbal working memory] and *q* = 0.788, 0.987, 0.437, 0.915, 0.431, and 0.126 [spatial working memory] at a respective channel). Regarding the oxy-Hb at the CH23 in the spatial working memory tasks at week 6 of the intervention, the two-way ANOVA showed statistically significant effects of interaction between group and time (*q* = 0.045, Table 3 and Figure 2). The changes of oxy-Hb in other channels, except for CH14, were higher in the β-lactolin than the placebo group in the verbal and spatial working memory tasks (Tables 2 and 3). There was no statistically significant difference in the oxy-Hb levels at week 0 of the baseline. The topography during the spatial working memory at week 6 of the intervention is shown in Figure 3. CH 23 was located at the left DLPFC area, thus, indicating that supplementation with β-lactolin increases the rCBF in the DLPFC during spatial working memory tasks. Conversely, there was no statistically significant difference in the oxy-Hb levels in the serial 7 subtractions (serial 7s) and rapid visual information processing (RVIP) tasks (data not shown).

**Table 2:** Oxy-Hb measurement in DLPFC during verbal working memory task.

	**Group**	**N**	**Week 0**	***q***	**Week 6**	***q***	**Δ**	**ANOVA q**
CH11	Placebo	24	0.50 ± 0.65	0.456	0.40 ± 0.66	0.991	-0.11 ± 1.09	0.493
	β-lactolin	24	0.19 ± 0.90		0.40 ± 0.75		0.21 ± 1.13	
CH13	Placebo	25	0.61 ± 0.89	0.701	0.82 ± 0.79	0.889	0.21 ± 1.18	0.493
	β-lactolin	23	0.49 ± 0.71		0.94 ± 1.17*		0.46 ± 0.92	
CH14	Placebo	23	-0.14 ± 0.94	0.456	0.04 ± 0.70	0.889	0.18 ± 1.23	0.493
	β-lactolin	24	0.13 ± 0.78		-0.02 ± 0.57		-0.15 ± 0.81	
CH20	Placebo	24	0.31 ± 1.37	0.701	0.34 ± 1.22	0.889	0.03 ± 2.07	0.72
	β-lactolin	24	0.15 ± 1.44		0.21 ± 1.16		0.06 ± 1.50	
CH22	Placebo	25	0.47 ± 0.75	0.456	0.63 ± 0.86	0.889	0.16 ± 1.03	0.493
	β-lactolin	24	0.09 ± 0.86		0.49 ± 0.73		0.40 ± 1.34	
CH23	Placebo	25	0.14 ± 0.65	0.456	0.10 ± 0.61	0.889	-0.05 ± 1.03	0.493
	β-lactolin	24	-0.05 ± 0.67		0.25 ± 0.73		0.30 ± 1.09	

The Z-score of Oxy-Hb measurement at weeks 0 and 6, and changes between weeks 0 and 6 in six channels (CH11, CH13, CH14, CH20, CH22, and CH23) during the verbal working memory task. Data are presented as means ± SD. The number of participants in each channel was represented. Group differences were identified using ANOVA and FDR correlation. Statistically significant differences between baseline and week 6 were identified using paired t-tests.

ANOVA, analysis of variance; CH, channel; Hb, hemoglobin; FDR, false discovery rate; DLPFC, dorsolateral prefrontal cortex

**Table 3:** Oxy-Hb measurement in the DLPFC during spatial working memory task.

	**Group**	**N**	**Week 0**	***q***	**Week 6**	***q***	**Δ**	**ANOVA *q***
CH11	Placebo	25	0.30 ± 0.61	0.788	0.09 ± 0.93	0.337	-0.20 ± 1.17	0.26
	β-lactolin	24	0.19 ± 0.57		0.48 ± 0.74		0.30 ± 0.85	
CH13	Placebo	25	0.47 ± 0.94	0.987	0.36 ± 0.98	0.337	-0.10 ± 1.22	0.26
	β-lactolin	23	0.47 ± 0.81		0.86 ± 1.17^†^		0.39 ± 0.99	
CH14	Placebo	23	-0.35 ± 0.87	0.437	-0.23 ± 0.93	0.543	0.12 ± 1.07	0.585
	β-lactolin	24	-0.03 ± 0.86		-0.04 ± 0.80		0.00 ± 0.99	
CH20	Placebo	24	0.00 ± 1.57	0.915	-0.16 ± 1.17	0.543	-0.16 ± 2.07	0.610
	β-lactolin	23	0.16 ± 2.06		0.14 ± 1.25		-0.02 ± 1.96	
CH22	Placebo	25	0.46 ± 0.74	0.431	0.54 ± 0.80	0.775	0.07 ± 0.95	0.452
	β-lactolin	24	0.15 ± 0.74		0.47 ± 0.82		0.32 ± 0.99	
CH23	Placebo	25	0.36 ± 0.81	0.126	0.18 ± 0.61	0.424	-0.17 ± 0.97	0.045
	β-lactolin	24	-0.22 ± 0.90		0.37 ± 0.37**		0.59 ± 0.94	

Oxy-Hb measurement at weeks 0 and 6, and changes between week 0 and week 6 in 6 channels (CH11, CH13, CH14, CH20, CH22 and CH23) during the spatial working memory task. Data are presented as means and ± SD. The number of participants in each channel was represented. Group differences were identified using ANOVA and FDR correlation. Statistically significant differences between baseline and week 6 were identified using paired t-tests. A value of *q* < 0.05 is considered statistically significant.

ANOVA, analysis of variance; CH, channel; Hb, hemoglobin; FDR, false discovery rate; DLPFC, dorsolateral prefrontal cortex.

### Cognitive tasks

Changes in task performance in the verbal and spatial working memory tasks during the rCBF measurement were shown in Supplementary Tables 1 and 2, respectively. Changes in the shortest reaction time in verbal working memory tasks showed a statistically significant reduction at week 6 of intervention in the β-lactolin group, compared with those at baseline (paired *t*-test; *p* = 0.023). This reduction was not observed in the placebo group (Supplementary Table 1). Changes in the average reaction time in spatial working memory tasks tended to be reduced at week 6 of intervention in the β-lactolin group, compared with those at baseline (paired *t*-test; *p* = 0.056) (Supplementary Table 2). Conversely, there were no statistically significant differences between the groups. The cognitive task levels are supposedly adequate to induce an rCBF increase with undetectable cognitive function differences, as the correct percentages in the verbal and spatial working memory tasks were more than 89–95 % (data not shown).

### Correlation between task performance and cerebral blood flow

The correlation between the change in the reaction time and the oxy-Hb levels at CH23 during the spatial working memory task is shown in the Supplementary Figure 1. We performed Spearman’s rank correlation for changes at 6 weeks to investigate the correlation between task performance and the left DLPFC’s activity during the spatial working memory task, which showed a statistically significant increase in the oxy-Hb levels. The reaction time and the oxy-Hb changes tended to be negatively correlated at CH23 (Spearman’s rank correlation; r = -0.27, *p* = 0.064) (Supplementary Figure 1) and showed a statistically significant negative correlation with those at CH 20 (Spearman’s rank correlation; r = -0.32, *p* = 0.030, data not shown). This result indicated that the left DLPFC’s oxy-Hb is positively correlated with task performance (reduction of average reaction time).

## DISCUSSION

This is the first study to demonstrate the effects of β-lactolin on the rCBF during cognitive tasks using a NIRS. Supplementation with β-lactolin increased the rCBF in the DLPFC area during working memory tasks, compared with the placebo group in a randomized, double-blind, placebo-controlled trial. These results suggested that supplementation with β-lactolin promotes DLPFC activity and improves the DLPFC-associated cognitive functions including memory retrieval, attention, and executive function.

The DLPFC is an area in the prefrontal cortex that plays an important role in the executive functions for the management of cognitive processes [22], such as working memory, cognitive flexibility [23], planning [24], inhibition, and abstract reasoning. Previous randomized controlled trials performed by our team demonstrated that supplementation with β-lactolin improved the executive function [14], selective and sustained attention [6], and associative working memory [6] in healthy older adults, as evaluated using the verbal fluency, Stroop, visual cancellation, and paired-associated tests, respectively. These functions are closely related to the function of the DLPFC. It is reported that damage to the DLPFC impairs working memory [25], attention [26], planning [27], motivation to perform self-improvement and social activities, and causes disinterest in their surroundings [28]. Dysfunction of the DLPFC is associated with schizophrenia [29], depression [30], stress [31], and Alzheimer’s disease [32]. Such dysfunction also reduced the rCBF in the DLPFC, especially its left side. It has been reported that rCBF activation was impaired in the left DLPFC and the number of task errors increased with aging, during the card sorting test in the elderly [33]. It has also been reported using SPECT that the rCBF in the left DLPFC is associated with full-scale intelligence quotient, perceptual reasoning, and processing speed in patients with moyamoya disease [34]. In addition, a report stated that the rCBF in the left DLPFC is required for the spatial rather than the verbal working memory in the elderly [35]. Taken together, it means that increased rCBF in the left DLPFC area produced by supplementation with β-lactolin is associated with improved cognitive function in older adults, as demonstrated in previous studies [6, 14]. Conversely, in this study, the rCBF in the DLPFC, during serial 7s and RVIP, was not changed by the supplementation of β-lactolin. Serial 7s and RVIP in the trial increased the rCBF in all area channels. In the z-score analysis, the rCBF at the DLPFC areas was relatively low and could not have been adequately evaluated. Serial 7s and RVIP tasks require attention associated with the function of the parietal and the frontal area. A report indicated that the RVIP task activates the rCBF in the fronto-parietal lobes in the experiment using positron emission tomography [36], potentially making it difficult to effectively evaluate the DLPFC’s activation by these tasks with the multi-channel NIRS. To conclude the mechanisms of neural activation by β-lactolin supplementation, further studies are needed to investigate the effects of β-lactolin on the DLPFC and in other fronto-parietal areas using adequate cognitive tasks.

Recent studies demonstrated that transcranial direct current stimulation to the DLPFC improves working memory, attention inhibition, and executive function in patients with Alzheimer’s disease [37–39]. Repeated stimulation to the left DLPFC using transcranial magnetic stimulation also improves depressive and anxious symptoms in medication-naïve patients with depression [40]. Conversely, such stimulations are required during surgery; however, they have not been approved as a therapy. Other non-invasive approaches to activate the DLPFC have gained increased attention. A recent study showed that working memory-training improves functional connectivity and the rCBF during rest in a clinical trial [41]. Some nutrients improve the rCBF in the frontal cortex in a clinical trial. For instance, it is reported that supplementation with Ginkgo biloba in older adults promotes rCBF and cognitive performance [42]. Conversely, there are limited approaches to promote rCBF in the DLPFC. Supplementation with β-lactolin will be a promising approach to promote rCBF in the DLPFC and improve cognitive function in the elderly.

The functions of the DLPFC are activated by neurotransmitters including dopamine, serotonin, norepinephrine, and Gamma-aminobutyric acid. Deficient neurotransmitters impair working memory, planning, inhibition, and decision making, especially dopamine, which is distributed mostly in the frontal cortex and is associated with its functions [43]. Aging reduces the dopamine levels in the frontal cortex, leading to cognitive decline [44]. Treatments that increase the dopamine levels improve cognitive function. The dopamine D1 receptors are mostly involved in working memory tasks [45]. Their levels in the frontal and parietal cortices are reduced by age and are associated with impaired working memory performance [46]. Additionally, in patients with Parkinson’s disease, L-dopa ameliorates high-level cognitive deficits by increasing the rCBF in the DLPFC, thus, suggesting that the dopamine system is involved in cognitive function improvement in the DLPFC [47]. Our previous preclinical studies demonstrated that oral β-lactolin administration activated the dopaminergic neurons and increased the dopamine levels in the cortex and the hippocampus, resulting in improved spatial working memory and object recognition memory in a rodent model. Orally administered β-lactolin is delivered to the brain through the blood-brain barrier and inhibits MAO-B, increases the dopamine levels, and improves impaired memory [5, 48]. The blockade of the dopamine D1 receptor attenuates the effects of β-lactolin to improve cognitive function in pharmacologically induced amnesiac mice. Moreover, our preclinical study showed that the dopamine levels in the cortex were reduced in Alzheimer’s disease model 5×FAD mice. Treatment with β-lactolin or Trp-Tyr of its core sequence increased the cerebral dopamine levels, reduced the cerebral amyloid β levels, and improved the impaired object recognition memory [8, 13]. These reports underscored the benefits of β-lactolin for improving the dopamine levels and increasing rCBF in the DLPFC via the dopaminergic system, thereby attenuating cognitive decline and dementia.

There were several limitations in this study. The rCBF in the left DLPFC area during the spatial working memory was significantly higher in the β-lactolin than in the placebo group but the verbal working memory was higher though not statistically significant. This trial indicated that β-lactolin supplementation activates the left DLPFC function; however, the area increased by β-lactolin was limited to the left side of the DLPFC (CH23). To conclude the effects of β-lactolin on the rCBF of the DLPFCs during working memory tasks, further studies are required to evaluate the effects of β-lactolin on the DLPFC activities during working memory tasks, involving a larger study population using NIRS, MRI, or SPECT.

In conclusion, this study is the first to demonstrate that supplementation with β-lactolin increases the rCBF in the left DLPFC area during working memory tasks. As such, supplementation with β-lactolin may be an effective approach to improve rCBF and promote cognitive function associated with the DLPFC, leading to prevention from dementia.

## MATERIALS AND METHODS

### Ethics and registration

The study was conducted in accordance with the Declaration of Helsinki and Ethical Guidelines for Medical and Health Research Involving Human Participants. Written informed consent was obtained from all participants. This trial was approved by the ethics committee of the Japanese Conference of Clinical Research (JCCR, Tokyo, Japan) and registered on the 11^th^ of January 2019 in the database of UMIN prior to enrollment (Registration No. UMIN000035521; Registration title. A study to evaluate the impact of test food ingestion on rCBF).

### Participants

We recruited 71 Japanese-speaking healthy older adults between 45 and 60 years of age (male/female = 35/36). Right-handed participants, as determined by the Japanese version of the Flinders Handedness survey (FLANDERS), were included [49]. Participants with minimal body movement during the rCBF measurement were preferentially included. The exclusion criteria were as follows: visual or hearing impediments, suspected dementia, anamnesis of cranial nerve disease, diagnosis of depression or depressive symptoms, menopausal symptoms or hormone treatment, irregular lifestyle (e.g., shift work), high consumption of alcohol (> 20 g/day), use of cigarettes, experience of the neuropsychological tests within 1 year, current treatment for cognitive functions, regular consumption of drugs or health functional supplements affecting cognitive functions including docosahexaenoic acids and gingko (> once a week), regular consumption of protein supplements (> once a week), anamnesis of severe disease requiring regular treatment, allergy or sensitivity to milk, and pregnancy or breastfeeding. The inclusion and exclusion criteria were checked during the screening steps using a questionnaire, Mini Mental State Examination (MMSE) (excluded if score ≤25), interviews by the principal investigator, and the data of rCBF measurement. Previous studies evaluating other functional ingredients, such as gingko, docosahexaenoic acids, and resveratrol, required 10–30 participants to detect statistically significant differences in rCBF (α = 0.05) [42, 50, 51]. To ensure sufficient statistical power after stratification, at least 25 participants were required per group.

### Experimental supplements

The test tablets containing 1.8 mg of β-lactolin in the whey enzymatic digestion were prepared by Kirin Holdings Co., Ltd (Tokyo, Japan) according to the previous study [6]. The amount of β-lactolin, one of the Trp-Tyr-related β-lactopeptides, was measured by the LC/MS/MS method using synthetic GTWY peptide (Peptide Institute, inc., Osaka, Japan) as a standard. The tablets were ingested every day for 6 weeks. As a previous work showed that supplementations with β-lactolin for 6 weeks improved the cognitive function, in we supplemented β-lactolin for this specific period. In placebo tablets, whey enzymatic digestions were substituted with the same amount of maltodextrin. The test and placebo tablets were indistinguishable by size, shape, and taste. The amount and periods of β-lactolin supplementation and tablet composition were equivalent to the previous trials because the current study aimed to investigate the mechanism of the previous findings [6, 14].

### Procedures

The trial was performed according to a randomized, placebo-controlled, double-blind, parallel-group comparative design. Questionnaires for the inclusion/exclusion criteria and lifestyle data, measurements of blood pressure, height, body weight, MMSE, rCBF, and medical interviews for safety assessments by a principal investigator were performed during the screening step. Selected participants were randomly allocated to the β-lactolin or placebo group with a computer program that ensured similar age, sex, MMSE, and oxy-Hb values between the study groups. The study group allocator was not involved in the assessment of eligibility, data collection, or analysis. The participants, research staff, and outcome assessors were blinded to group allocations until data analyses had been completed. During the intervention period, the participants were instructed to maintain regular lifestyles, avoid taking any drugs and new health functional supplements affecting cognitive function or CBF, and avoid taking protein supplements. Compliance was monitored using participant diaries. On the day of the rCBF measurement, the participants were instructed to completely avoid consuming caffeine and ingesting any foods and beverages, except for water, 2 h prior to the start of the tests. Data were collected at the Breast-health clinic (Tokyo, Japan) between January and March 2019. This study was conducted by the contract research organization, KT Medical Co., Ltd.

### Measurements of cerebral blood flow (Primary outcome)

The rCBF was measured using a 34CH NIRS system (WOT-HS34M, NeU Corporation, Tokyo, Japan), which used continuous wave laser diodes with two wavelengths (730 and 850 nm) as light sources and was able to detect concentration changes in oxy-Hb, deoxygenated hemoglobin (deoxy-Hb), and their sum (total-Hb). The transmitted light was detected every 100 ms with avalanche photodiodes located at 30 mm from the sources, forming 34 measurement channels. The probes of the NIRS were placed on the participants' frontal region, with the lowest probes being positioned along the Fp1-Fp2 line, as defined by the international 10–20 system used in electroencephalography. This probe arrangement can measure the Hb changes in the approximate surface regions bilaterally in the DLPFC (Brodmann’s areas 9 and 46), which was cover at CH11, CH13 and CH14 in the right side and CH20, CH22 and CH23 in the left side. The participants were fitted with the NIRS headset, of which the probe position was in accordance with the previous report, and the rules of tasks were explained to them as described below [52]. Moreover, the participants completed a 5-min rest period and, then, completed the task session. They performed eight, eight, one, and one trials of verbal working memory, spatial working memory, serial 7s, and RVIP tasks, respectively, as described in the following section, for a total of approximately 20 min (Supplementary Figure 2). Each session was separated by a short break (approximately 30 s). At weeks 0 and 6 of the intervention, the procedures measuring rCBF were performed in the same way. Τhe participants were seated in a comfortable chair in a quiet room and instructed to maintain this position during the tasks.

### Data analysis of cerebral blood flow

Analyses were performed using a plug-in-based analysis software, the platform for optical topography analysis tools (developed by Hitachi ARL.; run on MATLAB, The MathWorks, Inc., Natick, MA, USA). Oxy-Hb was the most sensitive variable to concentration changes in the regional rCBF, and provided the strongest correlation with the BOLD signal among the three NIRS parameters [53, 54]. Thus, we analyzed the changes in the oxy-Hb levels, as the best indicator of brain activity. In the present study, the changes of oxy-Hb were considered as those of the CBF. First, we calculated the relative values of concentration changes in oxy-Hb for each channel based on the modified Beer-Lambert law, using light signals transmitted at the two wavelengths. These signals were expressed as the product of the changes in Hb concentration (mM) and optical path length (mm) in the activation region (effective optical path length).

The NIRS system measured changes in the concentration of oxy-Hb and deoxy-Hb from the starting baseline. The baseline was obtained from the first (1.0) and the last (10.0) s of each block and was described in detail as follows. A Butterworth band-pass filter (0.02–0.80 Hz) was, then, applied to the oxy-Hb signals to remove low-frequency drift/oscillation and high-frequency system noise. In the verbal and spatial working memory tasks, the time-continuous data of Hb-signals were divided into 24.5-s task blocks, which consisted of a 1.0-s pre-task period (starting at 1.0 s before task period), an 8.5-s task period, and a 15.0-s post-task period (starting after task period). Similarly, in the serial 7s task, a set of 196.0 s task blocks, which consisted of a 1.0-s pre-task, a 180.0 s-task, and a 15.0-s post-task period. In the RVIP task, a set of 216.0-s task blocks, which consisted of 1.0-s pre-task, 200.0-s task, and 15.0-s post-task periods. Median filter methods were used to remove minor motion artifacts (window size, 2.0 s), and a baseline correction was performed using linear fitting based on two baseline periods: the first (1.0) s and the last (10.0) s of each block. For the verbal and spatial working memory tasks, individual time-course data for the oxy-Hb concentrations were averaged over the eight trials of each task. The data from each channel were converted into z-scores to reduce the impact of participant and channel differences and to compare appropriately [55]. The *z*-score was calculated using the mean and standard deviation of changes in the oxy-Hb concentrations. Consequently, the mean and the standard deviation were adjusted to a *z*-score of 0 and 1 for every channel, respectively. In this study, the ROI regarding the NIRS data were arranged into two regions: the right (CH 11, 13, and 14) and the left DLPFC area (CH 20, 22, and 23) [52, 56, 57]. Six channels were analyzed during the working memory tasks in the present study.

### Cognitive tasks

During measurements of rCBF, the participants performed four cognitive tasks: the verbal working memory, the spatial working memory [52, 58], the serial subtraction, and the RVIP [59, 60]. Before each task, the participants performed short trials to become familiarized with the procedures.

The verbal and the spatial working memory tasks were presented through a stimulus presentation system (SP-POST01; NeU Corporation, Tokyo, Japan), which provided an identical delayed-response paradigm (Supplementary Figure 3). In both tasks, each trial started with a 1.5 s presentation of the target stimuli (S1), which was followed by a 7.0 s delay. A probe stimulus (S2) was, then, presented for 2.0 s or until the participants responded. The reset intervals between S2 and the next S1 were from 16 to 24 s. The mark of a cross was presented on the center of the screen during the reset intervals. In addition, a visual cue (changing the color of the fixation cross) was presented for 0.5 s prior to target stimulation (S1) and auditory cues (1000 and 800 Hz pure tones of 100 ms duration) were presented prior to S1 and S2.

In the verbal working memory, four Japanese characters in Hiragana were presented as S1 in Supplementary Figure 2, and a Japanese character in Katakana was presented as S2 in Supplementary Figure 2. The participants were instructed to judge whether the character presented as S2 corresponded to any of the characters in S1 and, then, to press the appropriate button as quickly as possible. As the characters in S1 and S2 were presented in different Japanese morphograms (i.e., Hiragana/Katakana), the participants were prompted to make their judgments based on the phonetic information of the characters, not by their form. The task was scored for the shortest reaction and the average reaction time.

In the spatial working memory task, S1 was the location of four red squares out of eight locations, and S2 was the location of a red square (Supplementary Figure 2). The participants decided whether the red location presented in S2 was included in the S1. The shortest and average reaction times were recorded.

The serial subtraction and the RVIP task were presented on a touchpad using the Computerized Mental Performance Assessment System software (Northumbria University, Newcastle, UK). In the serial 7s, the participants had to keep subtracting 7 from a random starting number between 800 and 999 as quickly as possible. The starting number was cleared by the entry of the first response, and the three-digits thereafter were displayed as asterisks. The task’s duration was 3 min and was scored for the number of correct and total responses. In RVIP, the participants monitored a continuous series of single digits to respond to three consecutive odds or even digits as quickly as possible, which were projected on the screen at a rate of 100/min in a pseudo-random order for 3 min. The task was scored for the correct answer rate and the average reaction time.

### Statistical analysis

Statistical analyses of the rCBF were performed for six channels of the ROI using the R statistical software (R Foundation for Statistical Computing, Vienna, Austria). Statistical significance of the rCBF and task performance were evaluated by a mixed-design two-way ANOVA with a between-participant factor of treatment (β-lactolin and placebo) and a within-subject factor of period (pre and post). We used the false discovery rate (FDR) method to correct for multiple comparisons [61] with a threshold of FDR (*q*) < 0.05. Statistical analyses of other data were performed using SAS 9.4 (SAS Institute Inc., Cary, NC, USA) and differences between the groups were identified using unpaired t-tests. The comparisons of the test period (pre and post) were examined with the paired t-test. To evaluate the correlations between the oxy-Hb signal changes and task performance, Spearman’s rank correlation was performed according to the previous report [62]. Differences were considered statistically significant when *p* < 0.05 or *q* < 0.05.

## Supplementary Material

Supplementary Figures

Supplementary Tables
