# Supplementary material for: β-lactolin increases cerebral blood flow in dorsolateral prefrontal cortex in healthy adults: a randomized controlled trial
Source: Aging (Albany NY). 2020 Sep 29;12(18):18660–75. doi: 10.18632/aging.103951 (PMC7585116; doi:10.18632/aging.103951)
Supplement: Supplementary Figures [file aging-12-103951-s002..pdf]

SUPPLEMENTARY FIGURES

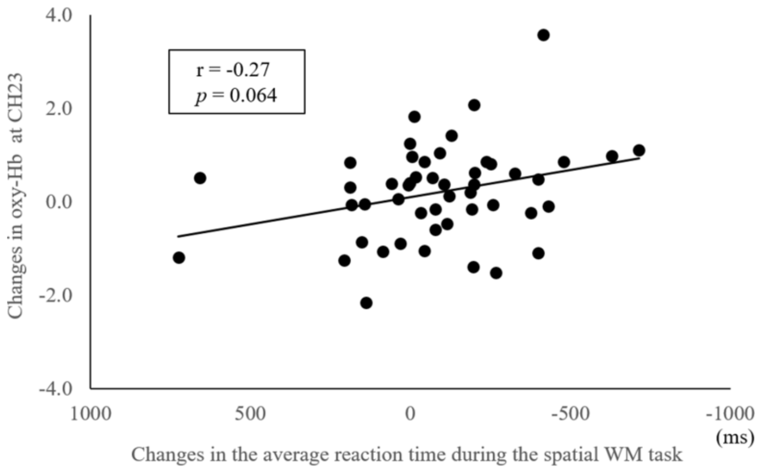

Supplementary Figure 1. Correlation between task performance and rCBF. Scatter plot showing correlation between the changes in average reaction time during the spatial working memory task and the changes in oxy-Hb at CH23 during the task. r: Spearman's rank correlation coefficient. *P*-values were calculated using Spearman's rank correlation. rCBF, regional cerebral blood flow.

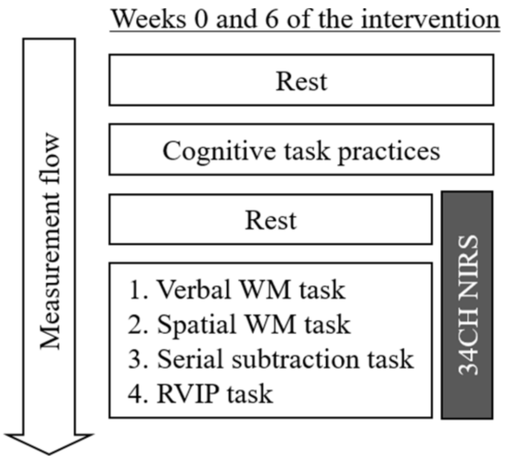

Supplementary Figure 2. Measurement flow. rCBF during working memory tasks was measured using a 34CH NIRS. CH, channel; NIRS, near-infrared spectroscopy; rCBF, regional cerebral blood flow; RVIP, rapid visual information processing.

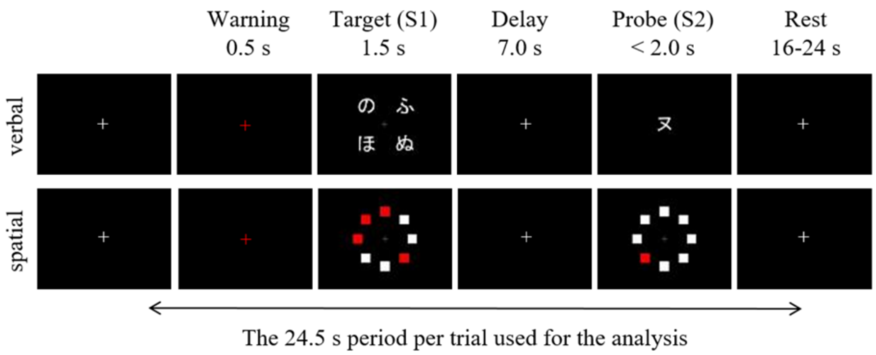

Supplementary Figure 3. Schematic diagram of the verbal and spatial working memory tasks. Images for the verbal and spatial working memory tasks are shown. The 24.5-s periods between the arrows were used for the analysis.
