# Supplementary material for: β-lactolin increases cerebral blood flow in dorsolateral prefrontal cortex in healthy adults: a randomized controlled trial
Source: Aging (Albany NY). 2020 Sep 29;12(18):18660–75. doi: 10.18632/aging.103951 (PMC7585116; doi:10.18632/aging.103951)
Supplement: Supplementary Tables [file aging-12-103951-s001..pdf]

SUPPLEMENTARY TABLES

Supplementary Table 1. Reaction time in verbal working memory task.

	Group	Week 0	*p*	Week 6	*p*	Δ	ANOVA *p*
Average reaction time (ms)	Placebo	1415.9 ± 301.1	0.915	1408.7 ± 320.8	0.488	-7.3 ± 255.1	0.391
	β-lactolin	1426.1 ± 356.5		1348.7 ± 276.4		-77.4 ± 310.0	
Shortest reaction time (ms)	Placebo	1018.4 ± 190.2	0.455	1007.7 ± 215.3	0.69	-10.7 ± 176.4	0.165
	β-lactolin	1060.5 ± 201.0		982.5 ± 224.6 *		-78.0 ± 156.8	

Average and shortest reaction time during the verbal working memory task. Data are presented as means ± SD for the placebo (n = 25) and the β-lactolin groups (n = 24). Group differences were identified using ANOVA. Time differences were identified using paired *t*-tests; *$p < 0.05$. ANOVA, analysis of variance; SD, standard deviation.

Supplementary Table 2. Reaction time in spatial working memory task.

	Group	Week 0	*p*	Week 6	*p*	Δ	ANOVA *p*
Average reaction time (ms)	Placebo	1571.0 ± 196.4	0.645	1507.3 ± 197.3	0.244	-63.7 ± 242.1	0.466
	β-lactolin	1539.5 ± 273.7		1419.7 ± 311.6 †		-119.9 ± 291.7	
Shortest reaction time (ms)	Placebo	1104.9 ± 213.0	0.836	1036.8 ± 195.8	0.817	-68.1 ± 196.0	0.979
	β-lactolin	1091.7 ± 233.2		1021.6 ± 259.1		-70.1 ± 323.4	

Average and shortest reaction times during the spatial working memory task. Data are presented as means and ± SD for the placebo (n = 25) and β-lactolin groups (n = 24). Group differences were identified using ANOVA. Time differences were identified using paired *t*-tests; † $p < 0.1$. ANOVA, analysis of variance; SD, standard deviation.
